# Integrated multi-omics analysis reveals the involvement of the gut-brain axis in children with autism

**DOI:** 10.3389/fmicb.2026.1766850

**Published:** 2026-02-04

**Authors:** Hongping Zhong, Shuyue Zhang, Zichao Mou, Xiaojing Fan, Xiayue Zhang, Lixia Wang, Xijia Xu, Xinxin Xue, Fan Yang, Jianbo Shu, Mingbang Wang, Chunquan Cai

**Affiliations:** 1 Clinical School of Pediatrics, Tianjin Medical University, Tianjin, China; 2 Department of Pediatrics, Yan'an University Affiliated Hospital, Yan'an, China; 3 Tianjin Children's Hospital (Children's Hospital of Tianjin University), Tianjin, China; 4 Department of Neonatology, Affiliated Shenzhen Women and Children's Hospital (Longgang) of Shantou University Medical College (Longgang District Maternity and Child Healthcare Hospital of Shenzhen City), Shenzhen, Guangdong, China; 5 Tianjin Pediatric Research Institute, Tianjin, China; 6 Tianjin Key Laboratory of Birth Defects for Prevention and Treatment, Tianjin, China

**Keywords:** 16S rRNA, autism spectrum disorder (ASD), gut-brain axis, mucin biosynthesis, multi-omics

## Abstract

**Background:**

Autism Spectrum Disorder (ASD) is frequently accompanied by gastrointestinal (GI) comorbidities and gut microbiota dysbiosis. While the microbiota-gut-brain axis is implicated in ASD pathophysiology, the upstream host genetic factors that drive these specific microbial alterations remain poorly characterized.

**Methods:**

To bridge this gap, we performed an integrated multi-omics analysis combining whole-exome sequencing, 16S rRNA gene sequencing, and plasma metabolomics in a cohort of children with ASD and typically developing controls.

**Results:**

We confirmed that children with ASD exhibit significant gut microbial dysbiosis and metabolic perturbations, which correlated with GI symptom severity. Crucially, rare variant enrichment analysis identified a significant accumulation of deleterious variants in mucin biosynthesis pathways (specifically the MUC gene family), which are essential for intestinal mucus barrier integrity. Multi-omics integration revealed that these host genetic defects were associated with distinct shifts in the gut ecosystem, notably the depletion of beneficial butyrate-producing bacteria (e.g., Faecalibacterium) and the expansion of mucin-degrading taxa. This structural dysbiosis translated into functional metabolic impairments, particularly in lipid transport and short-chain fatty acid metabolism, which tracked with ASD severity.

**Conclusion:**

Collectively, our data argue for a host-centric cascade where genetic vulnerabilities-specifically within the MUC pathway-compromise mucosal integrity, acting as a selective filter that fundamentally reshapes the gut microbiome. By pinpointing these variants as upstream drivers of gut-brain axis dysfunction, we move beyond simple association to identify concrete genetic targets-rare deleterious variants in the mucin (MUC) gene family-for future precision interventions in ASD.

## 1 Introduction

Autism Spectrum Disorder (ASD) is a severe neurodevelopmental condition that significantly impacts children’s health. Since Leo Kanner first described ASD in 1943, its prevalence has climbed to 1–2% globally, reflecting both improved detection and environmental shifts (Sun et al., 2019; Maenner et al., 2023; Zeidan et al., 2022; Croen et al., 2015). Typically emerging by age 3 with a notable 4:1 male predominance, ASD presents a challenge of immense clinical heterogeneity (First, 2013). In practice, we observe that patients rarely suffer solely from core social and behavioral deficits; rather, they are frequently burdened by comorbidities ranging from epilepsy to metabolic abnormalities, and autonomic nervous system dysregulation (Croen et al., 2015; Ellenbroek and Sengul, 2017). This complex clinical picture imposes a severe strain not only on the children’s physical health but also on their families, necessitating a deeper investigation into its underlying mechanisms.

Mounting evidence suggests that ASD is a complex disease driven by the interplay of genetic and environmental factors (Chaste and Leboyer, 2012). Extensive research has confirmed that genetic factors play a predominant role in the etiology of ASD, with heritability estimated at approximately 81%, involving both rare and common variants (Trost et al., 2022; Grove et al., 2019; Sandin et al., 2017). Recently, the “microbiota-gut-brain axis” has emerged as a critical environmental factor in the development of neurodevelopmental disorders (Sherwin et al., 2016; Cryan et al., 2019). In clinical practice, we frequently observe that children with ASD are burdened by gastrointestinal distress, such as constipation and abdominal pain, pointing to underlying disturbances in the gut microenvironment (Lou et al., 2022). Since gut maturation parallels early neurodevelopment, these microbial deviations are not merely comorbidities but are increasingly seen as drivers of symptom severity (Xie et al., 2022; Wan et al., 2022).

**Figure 1:**
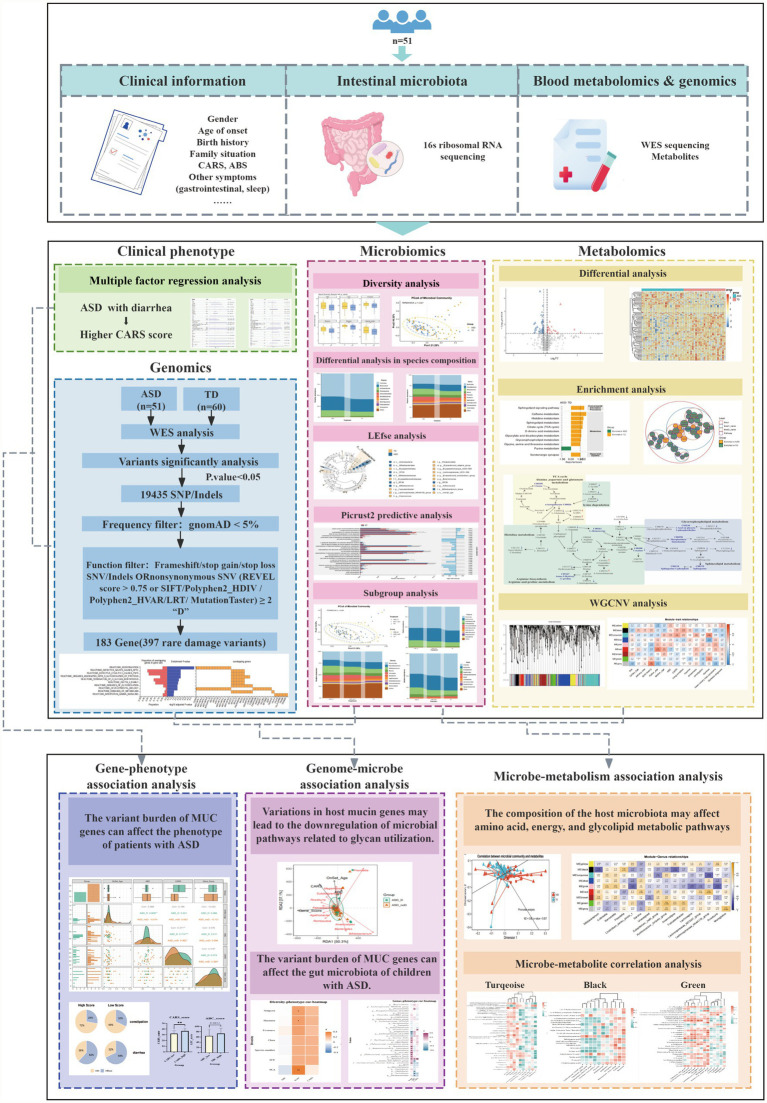
Schematic diagram of integrated multi-omics data analysis workflow for children with ASD. This figure illustrates the integrated multi-omics analysis framework established in this study to investigate the core mechanisms of gut-brain axis dysfunction in children with ASD, encompassing clinical phenotypes, genomics, microbiomics, and metabolomics.

Current high-throughput technologies, such as whole-exome sequencing (WES) and metabolomics, have revolutionized clinical screening and diagnosis. However, relying on a single omics layer often fails to capture the massive heterogeneity of ASD (Veenstra-Vanderweele et al., 2004). To bridge this gap, we adopt a systems biology perspective: does the host’s genetic background reshape gut ecology, and do specific microbial metabolites conversely influence brain function? By integrating 16S rRNA sequencing with metabolomics and genomics data (Figure 1), this study seeks to map these multidimensional “microbe-metabolite” interactions, offering novel insights into how the gut microenvironment modulates the variable phenotypes seen in ASD.

## 2 Methods

### 2.1 Data collection

A total of 51 children with ASD and 51 age-matched typically developing (TD) controls were recruited for this study. To ensure diagnostic rigor, inclusion for the ASD group relied on independent confirmation by two pediatric specialists adhering to DSM-5 criteria (Regier et al., 2013), with behavioral phenotypes further corroborated using the Childhood Autism Rating Scale (CARS) and Autism Behavior Checklist (ABC). The two groups were well matched for age and gender ratio with no significant differences (Supplementary Tables S1, S2). Conversely, the control group included neurotypical children cleared during routine developmental screenings. To minimize potential confounding effects and ensure cohort homogeneity and comparability, strict exclusion criteria were applied: (1) history of antibiotic use within 1 month prior to sampling; (2) history of probiotic supplementation within 2 weeks prior to sampling; (3) comorbidity with epilepsy or other neurological disorders; and (4) history of severe gastrointestinal (GI) organic diseases (e.g., Hirschsprung’s disease, inflammatory bowel disease). The diagnostic criterion for diarrhea is that the diagnosis can be made based on the parents’ or caregivers’ chief complaints regarding changes in the child’s stool characteristics (presenting as watery stools, pasty stools, or mucopurulent stools) and an increase in the number of bowel movements compared with usual (Subspecialty Group of Infectious Diseases et al., 2021). For all participants, detailed clinical and demographic data were collected, including biological sex, age, geographic origin, and family history. The genetic background consistency of the enrolled subjects was strictly controlled. All participants were of the same ethnic origin and had no consanguineous relationships, so as to minimize the confounding effects of population genetic structure differentiation.

### 2.2 Genomic analysis

The genomic characterization employed complementary sequencing methodologies: Sanger sequencing for targeted variant confirmation alongside comprehensive WES for broad variant detection. Peripheral venous blood samples (2 mL) were collected from each patient and their parents using EDTA anticoagulant tubes. Detailed experimental and bioinformatic protocols for Sanger sequencing and WES are provided in the Supplementary materials.

To identify genetic risk loci associated with ASD, we recruited 60 neurotypical individuals as the control group. Fisher’s exact test was employed to detect variants showing significant frequency differences between cases and controls (*p* < 0.05). A series of stringent annotation and filtering criteria were then applied to these differential variants. First, only variants located within protein-coding exons were retained, while those in intronic, UTR, regulatory, or intergenic regions were excluded. Second, we focused on rare variants with potential functional consequences. The inclusion criteria were as follows: (1) protein-truncating variants, including frameshift, nonsense, or stop-loss SNVs/Indels; or (2) missense SNVs predicted to be deleterious (REVEL score > 0.75 or classified as “damaging” by at least two of the following tools: SIFT, PolyPhen2_HDIV, PolyPhen2_HVAR, LRT, and MutationTaster) (Wang et al., 2010). A combined multi-algorithm prediction strategy can effectively reduce the false positive rate in variant pathogenicity prediction. All variants retained for the final analysis had a population frequency of less than 5% in the gnomAD database (Richards et al., 2015). To capture a broad spectrum of genetic susceptibility, the mode of inheritance was not applied as a filtering criterion in our analysis. Finally, functional enrichment analysis of the candidate genes was performed using the GENE2FUNC tool implemented in FUMA (Watanabe et al., 2017).

### 2.3 16s rRNA sequence

Fecal samples from children with autism spectrum disorder (ASD) and matched controls were collected in sterile containers from fasting participants in the early morning, and rapidly stored at −80 °C within 2 h. Throughout the entire sample processing procedure, repeated freeze–thaw cycles, exposure to room temperature, and environmental contamination were strictly avoided to preserve the integrity of microbial community structures. Detailed protocols for DNA extraction, library construction, and initial quality filtering of raw data are provided in the Supplementary methods.

Statistical analysis were conducted in R, with data visualization performed using the ggplot2 (v4.0.0) and LorMe package (v2.0.1). Our approach initially profiled the broad microbial architecture, rigorously comparing community composition alongside alpha- and beta-diversity metrics. To move from general patterns to specific drivers, we applied Linear Discriminant Analysis Effect Size (LEfSe) to isolate taxonomic biomarkers distinguishing the ASD cohort. Finally, shifting focus from taxonomy to potential metabolism, we inferred the functional capacity of the microbiome using the PICRUSt2 algorithm.

### 2.4 Metabolic analysis

For metabolomic profiling, this study included 25 ASD patients along with 25 healthy controls. Peripheral blood samples from the ASD patients underwent centrifugation followed by storage at −80 °C. A 100 μL sample was mixed with four volumes of methanol by vortexing, then incubated for 60 min. The sample was then centrifuged at 4 °C (3,000 r/min, 20 min), and the final supernatant was collected for lyophilization. Non-targeted metabolomics analysis was performed by Beijing Genomics Institution. Raw data were obtained after metabolite extraction, LC–MS/MS detection and quality control (Supplementary methods).

Metabolomic data processing involved peak extraction, quantification, and database matching identification through Compound Discoverer 3.1. Differential metabolites were screened using multivariate statistical analysis such as PCA and PLS-DA, followed by pathway enrichment analysis conducted in ReportScore.

WGCNA was performed with the “WGCNA” R package on the top 1,000 most variable metabolites (Langfelder and Horvath, 2008). An adjacency matrix was converted into a Topological Overlap Matrix (TOM) for module identification. Using average linkage hierarchical clustering on TOM-based dissimilarity, we grouped metabolites into modules via the dynamic tree cut algorithm (minimum size = 30). Only modules demonstrating significant correlations (*p* < 0.05) were retained for further analysis. In WGCNA, co-expression modules are identified and assigned color labels (e.g., Turquoise, Blue, Brown) by default to distinguish modules. These color names are arbitrary identifiers generated by the algorithm and do not have inherent biological meaning.

### 2.5 Integrated multi-omics analysis

Integrated multi-omics analysis was performed using R. Procrustes analysis was applied to assess the concordance between gut microbiome (genus level) and metabolome (differentially abundant metabolites) profiles. Bray–Curtis dissimilarity matrices were generated for both datasets and dimensionally reduced using Non-metric Multidimensional Scaling (NMDS). The correspondence between the two datasets was evaluated using the Procrustes function in the vegan package, with statistical significance determined via the PROTEST permutation test (10,000 permutations). Additionally, Distance-based Redundancy Analysis (dbRDA) based on Bray-Curtis distance was conducted to visualize multivariate associations. Additionally, Spearman’s rank correlation was employed to evaluate pairwise associations among microbial taxa, metabolic features, host genetic factors, and clinical phenotypes.

### 2.6 Statistical analysis

All statistical analyses were performed using R version 4.3.2 (R Project for Statistical Computing). To identify significant divergences between cohorts, we applied the non-parametric Mann–Whitney U test, treating *p*-values < 0.05 as statistically meaningful.

## 3 Results

### 3.1 Cohort characteristics

A total of 51 children with ASD were enrolled in this study, including 36 boys (70.6%) and 15 girls (29.4%). The median age at onset was 2.2 years. A detailed review of perinatal records revealed a spectrum of early-life adverse events, most notably umbilical cord entanglement (17.6%) and macrosomia (11.8%), alongside instances of aspiration (10.0%) and low birth weight (8.0%). Rare complications such as placental abnormalities (2.0%) and neonatal asphyxia (2.0%) were also documented. Clinically, the disease phenotype extended well beyond neurodevelopment: sleep architecture was frequently disrupted (insomnia: 35.3%; hypersomnia: 21.6%), and somatic comorbidities were striking. In particular, a vast majority struggled with constipation (70.6%) and lower urinary tract symptoms (41.2%), and this prevalence of gastrointestinal symptoms is consistent with the incidence reported in previous epidemiological studies of the ASD population (Holingue et al., 2018), underscoring the pervasive, multisystemic nature of the disorder (Table 1).

**Table 1:** Clinical manifestations of 51 children with ASD.

Category	Subcategory	*n* (%)/Mean (range)
Demographics	Gender	
	Male	36 (70.6%)
	Female	15 (29.4%)
	Age at onset (years)	2.23 (1.59–2.34)
Birth history	Gestational age	
	<37 weeks (Preterm)	2 (4%)
	>42 weeks (Postterm)	2 (4%)
	37–42 weeks (Term)	47 (92%)
	Mode of delivery	
	Vaginal delivery	29 (56.9%)
	Cesarean section	22 (43.1%)
	Adverse birth history	
	Macrosomia (4,000 g)	6 (11.8%)
	Low birth weight (2,500 g)	4 (8%)
	Umbilical cord entanglement	9 (17.6%)
	Amniotic fluid/meconium aspiration	5 (10%)
	Placental abnormalities	1 (2%)
	Postnatal asphyxia	1 (2%)
Family background	Parental age at pregnancy (years)	
	Father	33.67 (31–34)
	Mother	29.22 (27–31)
	Residential area	
	Urban	43 (84.3%)
	Town	5 (10%)
	Rural	3 (6%)
	Annual household income (RMB)	
	<50,000	21 (41.2%)
	50,000–100,000	25 (49%)
	100,000–500,000	5 (10%)
	Father’s educational level	
	Junior high school or below	18 (35.3%)
	Senior high school/Technical secondary school	14 (27.5%)
	Junior college	15 (29.4%)
	Bachelor’s degree	3 (6%)
	Master’s degree or above	1 (2%)
	Mother’s educational level	
	Junior high school or below	18 (35.3%)
	Senior high school/Technical secondary school	12 (23.5%)
	Junior college	6 (11.8%)
	Bachelor’s degree	12 (23.5%)
	Master’s degree or above	3 (6%)
Behavioral phenotype	Repetitive and restrictive behaviors	42 (82.4%)
	Oppositional defiant disorder	45 (88.2%)
	Sleep disorders	
	Insomnia	18 (35.3%)
	Hypersomnia	11 (21.6%)
Other clinical phenotypes	Gastrointestinal disorders	
	Constipation	36 (70.6%)
	Diarrhea	18 (35.3%)
	Lower urinary tract symptoms	21 (41.2%)

**Figure 2:**
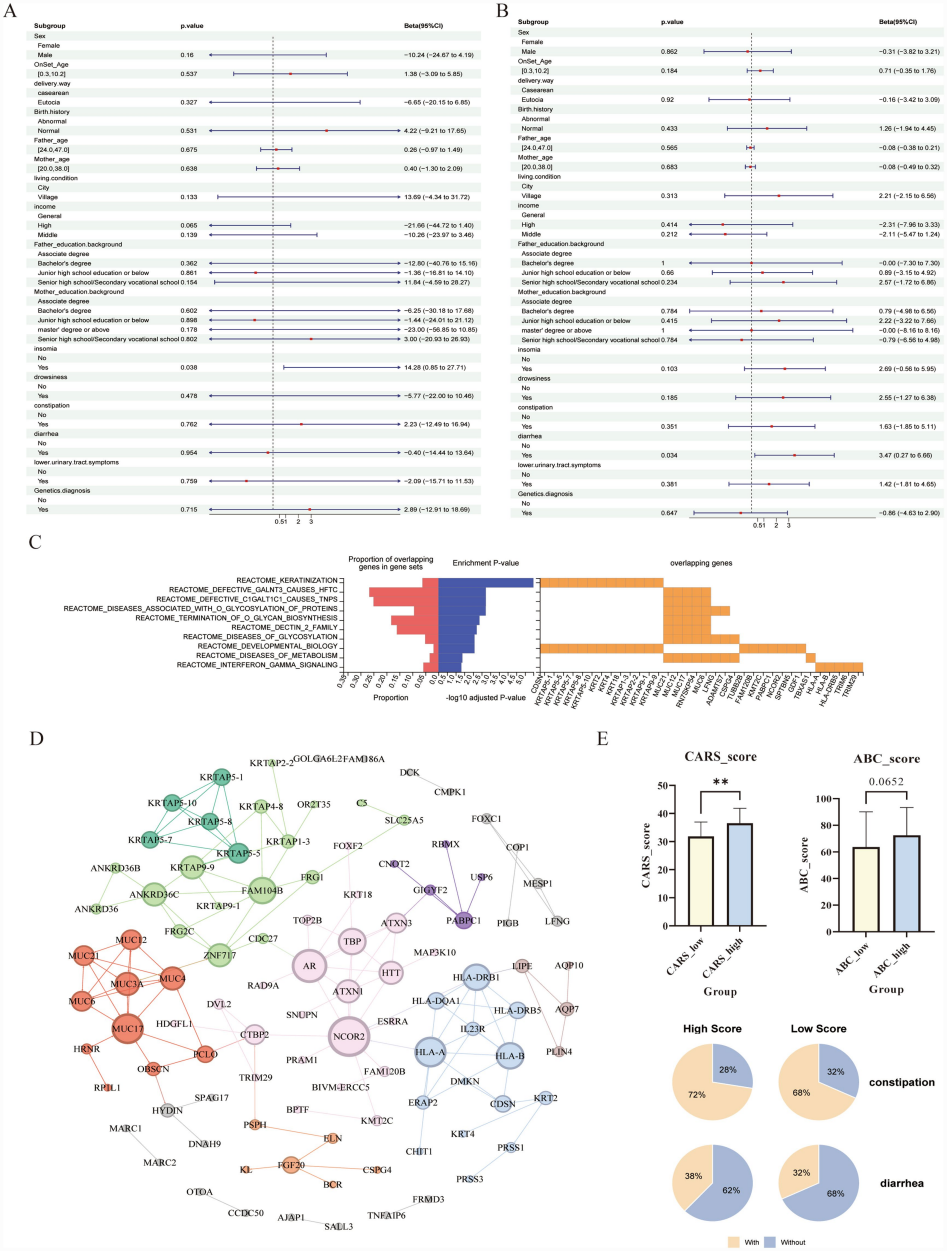
Clinical association analysis and identification of MUC pathway variants as key drivers in ASD. **(A,B)** Forest plots illustrating the multivariate regression analysis of clinical characteristics associated with **(A)** ABC total scores and **(B)** CARS scores. Key significant associations are highlighted with *p*-values <0.05. **(C)** Pathway enrichment analysis of rare deleterious variants. The left panel displays the significance (−log10 *p*-value, blue bars) and gene overlap proportion (orange bars) of the top enriched pathways. The right panel visualizes the gene-pathway matrix. **(D)** Protein–protein interaction (PPI) network of identified genes constructed using the STRING database. The network is clustered into functional modules, with the red cluster representing the mucin-related gene family. **(E)** Phenotypic consequences of MUC variant burden. Top: Comparison of CARS and ABC scores between groups with low and high MUC pathway variant burden scores. Data are presented as mean ± SEM (**p < 0.01; *p*-value for ABC score is indicated). Bottom: Pie charts showing the differential prevalence of gastrointestinal symptoms (constipation and diarrhea) between the high and low MUC variant burden groups.

Multivariate regression analysis revealed that the presence of diarrhea was significantly associated with higher CARS scores (*β* = 3.47, 95% CI: 0.27–6.66, *p* < 0.05). In the ABC model, insomnia in children was associated with significantly elevated ABC total scores (*β* = 14.82, 95% CI: 0.85–27.71, *p* = 0.038). Additionally, there was also a pattern in which children from high-income families tended to have lower ABC scores (*β* = −21.66, 95% CI: −44.72–1.40, *p* = 0.065), with a similar but weaker trend observed among those from middle-income families (*β* = −10.26, 95% CI: −23.97–3.46, *p* = 0.139). However, not all income-related differences reached statistical significance, and this trend requires further validation in larger cohorts (Figures 2A,B).

### 3.2 Genomic analysis results

We performed trio-based whole-exome sequencing (Trio-WES) on 51 ASD probands and identified pathogenic variants in 12 patients (23.5%) (Table 2).

**Table 2:** Clinically interpretable pathogenic variants identified by WES in 51 children with ASD.

No.	Sex	Type	Gene	Variants	Source
A3	Female	Nonsynonymous variants	*NBEA*	c.8287G > A (p.Gly2763Arg)	de novo
A4	Female	Nonframeshift variants	*ZBTB20*	c.1781_1786dup (p.Lys595_His596insLeuLys)	de novo
A5	Female	Nonsynonymous variants	*CSDE1*	c.896 T > C (p.Phe299Ser)	de novo
A6	Male	Frameshift variants	*UBE3A*	c.2567_2568del (p.Lys856ArgfsTer24)	de novo
A11	Female	Nonsynonymous variants	*KMT5B*	c.914G > T (p.Cys305Phe)	de novo
A14	Male	Frameshift variants	*SMARCE1*	c.9_12del (p.Lys3AsnfsTer67)	de novo
A17	Female	Nonsynonymous variants	*SETD1A*	c.1780C > T (p.Pro594Ser)	de novo
A20	Female	Nonsynonymous variants	*CHD7*	c.5734C > A (p.Arg1912Ser)	de novo
A23	Male	Frameshift variants	*PRRT2*	c.649dup (p.Arg217ProfsTer8)	Maternal variants
A39	Male	Nonsynonymous variants	*BCL11A*	c.7C > G (p.Arg3Gly)	de novo
A43	Male	Nonsynonymous variants	*CAMK2A*	c.775C > T (p.Arg259Cys)	de novo
A49	Female	Nonsynonymous variants	*SETD2*	c.6917A > G (p.Tyr2306Cys)	de novo

The susceptibility loci analysis identified 183 genes carrying rare damaging variants (comprising 397 variants) (Figure 2D; Supplementary Table S3). Enrichment analysis of these genes showed significant clustering in several functional pathways, including developmental signaling (e.g., Notch), immune-inflammation (e.g., interferon-gamma), glycosylation (O-glycosylation), and cell barrier function (e.g., keratinization) (Figure 2D). This result suggests that, in addition to directly affecting neurodevelopment, genetic factors may also interfere with neural networks via immune- and glycosylation-mediated signaling pathways, contributing to the ASD phenotype (Figure 2C).

We found *MUC21*, *MUC12*, *MUC17*, and *MUC6* genes enriched in O-glycosylation and Dectin-2 family pathways (Table 3). We calculated the rare damaging variant burden in these pathways and stratified patients by the median. The group with a high mutation burden exhibited a tendency toward more frequent gastrointestinal (GI) symptoms, with a significantly higher Childhood Autism Rating Scale (CARS) score (*p =* 0.0022) (Figure 2E). These findings suggest that the cumulative rare damaging variant burden in pathways such as O-glycosylation and Dectin-may 2 be a key genetic factor linking gastrointestinal dysfunction with a more severe ASD phenotype.

**Table 3:** Details of significant rare deleterious variants identified in MUC pathway genes in children with ASD.

Gene	Cytoband	Variant type	Transcript ID	cDNA change	Amino acid change	gnomAD freq	*P*-value	Predicted effect
*MUC12*	7q22.1	Non-frameshift insertion	NM_001164462	c.52delinsACTG	p.Thr18_Thr19 ins…	0.0023	3.68e-15	–
*MUC17*	7q22.1	Missense	NM_001040105	c.C2948A	p.Thr983Asn	0.0225	1.201e-05	Damaging
*MUC17*	7q22.1	Missense	NM_001040105	c.G3925A	p.Val1309Met	0.0312	0.00025	Damaging
*MUC6*	11p15.5	Missense	NM_005961	c.C5709G	p.Ser1903Arg	0	7.82e-05	Damaging
*MUC6*	11p15.5	Missense	NM_005961	c.C5521A	p.Pro1841Thr	1.878e-05	1.39e-14	Damaging
*MUC6*	11p15.5	Missense	NM_005961	c.T5507A	p.Leu1836His	5.531e-06	6.98e-17	Damaging
*MUC6*	11p15.5	Missense	NM_005961	c.C5272T	p.His1758Tyr	0	0.049	Damaging
*MUC6*	11p15.5	Missense	NM_005961	c.T4759C	p.Ser1587Pro	0.0065	3.65e-08	Damaging
*MUC21*	6p21.33	Frameshift insertion	NM_001010909	c.426delinsATGTG	p.Thr142fs*	0.0081	0.025	–

### 3.3 Aberrant gut microbial diversity patterns and taxonomic composition in ASD

**Figure 3:**
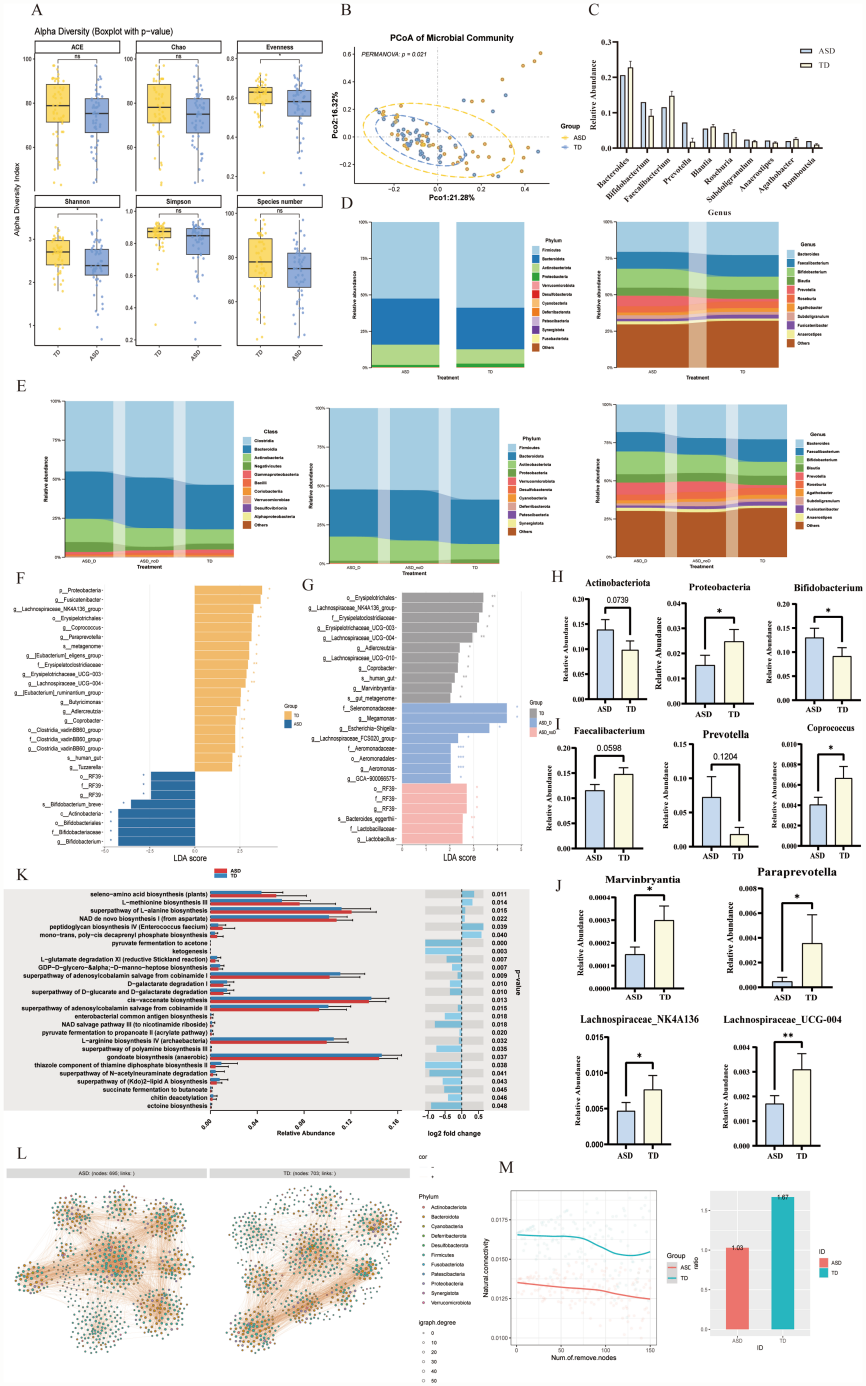
Structural dysbiosis, functional alterations, and impaired ecological stability of the gut microbiota in children with ASD. **(A)** Comparison of alpha diversity indices (ACE, Chao1, Evenness, Shannon, Simpson, and Species number) between the ASD and TD groups. Boxplots represent the median and interquartile range (Wilcoxon rank-sum test; ns, not significant; * *p* < 0.05). **(B)** Principal Coordinate Analysis (PCoA) based on Bray–Curtis dissimilarity. Ellipses indicate 95% confidence intervals. **(C)** Relative abundance of the top dominant genera in the ASD and TD groups. Data are presented as mean ± SEM. **(D)** Taxonomic composition profiles illustrating the relative abundance of major bacteria at the phylum (left) and genus (right) levels across the two groups. **(E)** Taxonomic composition profiles illustrating the relative abundance of major bacteria at the (A) phylum, (B) class, and (C) genus levels across the ASD with diarrhea (ASD_D), ASD without diarrhea (ASD_noD), and typically developing (TD) groups. **(F)** LEfSe identifying differentially abundant taxa. Histogram bars represent the LDA scores of biomarkers enriched in ASD (blue) and TD (yellow) groups (LDA threshold >2.0, *p* < 0.05). **(G)** LEfSe identifying differentially abundant taxa among the subgroups. Histogram bars represent the LDA scores of biomarkers enriched in ASD_D (blue), ASD_noD (pink), and TD (gray) groups (LDA threshold >2.0, *p* < 0.05). **(H–J)** Relative abundance comparisons of representative differentially abundant taxa at the **(H)** phylum and **(I,J)** genus levels identified across groups. **(K)** Functional prediction of microbial communities using PICRUSt2. The left panel shows relative abundance, and the right panel indicates the Log2 fold change and significance (*p*-value). **(L)** Co-occurrence network analysis. Visualizations of microbial interaction networks constructed for ASD (left) and TD (right) groups. Nodes represent genera, and edges represent significant correlations. The ASD network exhibits altered topology compared to the TD network. **(M)** Ecological stability analysis. Left: Robustness testing showing the decline in natural connectivity as nodes are sequentially removed (simulating species loss). The ASD network (red line) shows a faster decline in connectivity compared to the TD network (blue line), indicating lower stability. Right: Comparison of the ratio of negative correlations within the co-occurrence networks.

PCoA analysis of beta diversity revealed that the community structure within the ASD group was significantly more heterogeneous than in the TD group (*p_PERMANOVA_* = 0.021). In terms of alpha-diversity, the TD group exhibited significantly higher Shannon (diversity) and Evenness indices compared to the ASD group (*p_Shannon_* = 0.02113 and *p_Evenness_* = 0.04089). Concurrently, the TD group also showed a trend towards higher Simpson, ACE, and Chao1 indices. Together, these results indicate that the gut microbiota of TD control children has greater species diversity and community evenness than that of children with ASD (Figures 3A,B).

At the phylum level, taxonomic composition analysis showed that the abundances of *Proteobacteria* and *Patescibacteria* differed significantly between the ASD and TD groups. Furthermore, the ASD group exhibited clear trends in microbial shifts: a lower abundance of *Firmicutes* and higher abundances of *Bacteroidota* and *Actinobacteriota* compared to the TD children (Figures 3D,H; Supplementary Figure S1A). At the genus level, a significant imbalance was observed in the core microbiota: the beneficial genus *Faecalibacterium* and *Bacteroides* showed a decreasing trend. However, *Bifidobacterium* abundance was paradoxically increased, and *Prevotella* also exhibited an increasing trend (Figures 3C,D,I). Among rare or specific genera, *Aeromonas* was highly significantly enriched in the ASD group. Furthermore, *Coprococcus, Marvinbryantia, Adlercreutzia*, and *Paraprevotella* were also statistically different (*p* < 0.05). Notably, multiple genera within the *Lachnospiraceae family (UCG-004, UCG-010, NK4A136_group)* also differed, suggesting an alteration in the butyrate-producing bacteria community in children with ASD (Figure 3J; Supplementary Figure S1B).

LEfSe analysis was conducted to identify differential microbial taxa between the two groups, using an LDA score > 2 as the selection threshold. At the phylum level, Actinobacteriota was significantly enriched in the ASD group, whereas Bacteroidota and Firmicutes were predominantly enriched in the TD group. At the genus level, Bifidobacterium was markedly increased in children with ASD, while *Lachnospiraceae* and *Ruminococcus* were significantly elevated in the TD group (Figure 3F).

PICRUSt2 prediction revealed significant metabolic dysregulation in the ASD group (Figure 3K). Energy and amino acid metabolism were notably affected: ketogenesis (*p* = 0.003) and L-glutamate degradation (*p* = 0.007) were downregulated, while biosynthesis of L-alanine (*p* = 0.015) and L-methionine (*p* = 0.014) was enriched. Furthermore, pathways for carbohydrate digestion (*p* = 0.029) and riboflavin metabolism (*p* = 0.012) were enriched.

Based on our previous findings that linked diarrhea to higher CARS scores and genetic variations, we conducted a subgroup analysis on ASD patients with concomitant diarrhea. Alpha and beta diversity analysis indicated that the presence of diarrhea did not significantly affect the richness or evenness of the gut microbiota in ASD patients (Supplementary Figures S1C,D). We further performed a taxonomic composition analysis, which revealed a structural gradient at the Class level across the three groups (Figure 3E). At the genus level, Megamonas was identified as a distinctive biomarker for the ASD_D group by both Kruskal-Wallis and LEfSe analyses, co-enriched with opportunistic pathogens like Escherichia-Shigella. In contrast, the TD group was characterized by beneficial butyrate-producing genera, specifically Lachnospiraceae_NK4A136_group and UCG-004, whereas the ASD_noD group showed specific enrichment of Lactobacillus and RF39 (Figure 3G; Supplementary Figure S1E).

### 3.4 Co-occurrence network topology and ecological stability

To explore the fundamental ecological differences between the ASD and TD gut microbiota, we constructed co-occurrence networks and compared key topological parameters. Superficially, the ASD network appeared more robust, characterized by hyper-connectivity—exhibiting higher connectance (0.0181 vs. 0.0155), total edges (4,369 vs. 3,834), and average degree (12.57 vs. 10.91) relative to TD controls. Yet, this density appears maladaptive. Unlike the TD network, which was stabilized by a rich fabric of negative (competitive) interactions, the ASD network suffered from markedly lower natural connectivity. We interpret this dense yet inefficient architecture not as a sign of vigor, but of unregulated redundancy, rendering the community structurally brittle and prone to collapse under stochastic perturbations (Figures 3L,M).

### 3.5 Serum metabolomic analysis in ASD

**Figure 4:**
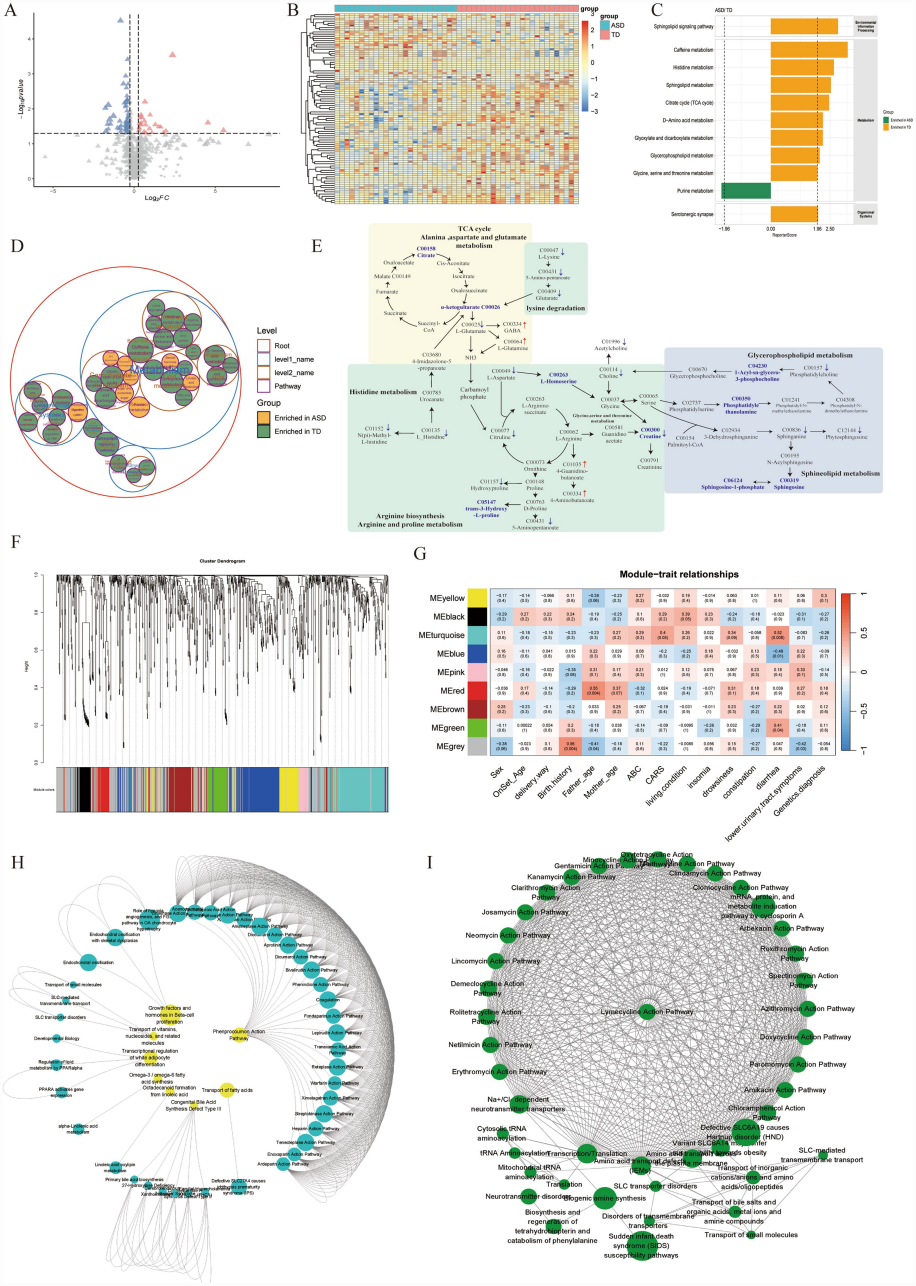
System-wide metabolic disturbances and identification of clinically relevant metabolite modules in ASD. **(A,B)** Volcano plot **(A)** and heatmap **(B)** of significant differentially metabolites. **(C,D)** Pathway enrichment analysis of differential metabolites: **(C)** Bar chart showing significant KEGG pathway enrichment, and **(D)** classification plot displaying the super-class distribution of altered metabolites. **(E)** A schematic diagram summarizing the interconnected dysregulated pathways. Bold text denotes significantly altered metabolites, and arrows indicate the direction of up- or down-regulation. **(F)** Cluster dendrogram from WGCNA. Metabolites were clustered into distinct co-expression modules, represented by different colors in the bottom bar. **(G)** Module-trait relationship heatmap. Each cell contains the Spearman correlation coefficient and *p*-value between a metabolic module (rows) and clinical traits (columns). **(H,I)** Network visualization of the key functional modules. Nodes represent metabolic pathways within the **(H)** turquoise module and **(I)** green module (representative modules), illustrating the internal connectivity and key driver pathways linked to clinical phenotypes.

Serum metabolomic profiling of our 25-pair cohort unveiled a distinct metabolic signature in ASD, characterized by 88 dysregulated metabolites (20 upregulated, 68 downregulated; Figures 4A,B). Enrichment analysis pointed to systemic disruptions in energy and lipid homeostasis, specifically affecting the TCA cycle, glycerophospholipids, and sphingolipids. Crucially, these perturbations extended into amino acid turnover (arginine/proline, lysine), intersecting directly with neurochemical signaling via the glutamate-GABA cycle and serine/glycine metabolism. This suggests that the observed metabolic shifts are not merely peripheral, but may echo deep-seated disruptions in the brain’s neurochemical architecture (Figures 4C–E).

To further investigate the relationships between plasma metabolites and clinical phenotypes in children with ASD, we performed WGCNA. Using the scale-free topology criterion, a soft-thresholding power beta was selected at an R^2^ of 0.85, resulting in the identification of 9 metabolite co-expression modules (Figure 4F). Module-trait correlation analysis revealed that 3 modules were significantly associated with the diarrhea phenotype. The turquoise module (r = −0.52, *p* = 0.008) and the green module (r = 0.41, *p* = 0.04) showed significant positive correlations with diarrhea, while the blue module (r = −0.49, *p* = 0.01) exhibited a significant negative correlation. Notably, the Turquoise cluster also trended toward a correlation with ASD severity (CARS scores; r = 0.40, *p* = 0.05), hinting at a potential biological link between gut metabolic state and behavioral intensity (Figure 4G).

Biological annotation of these trait-associated modules offered mechanistic clues. The Blue module was heavily anchored in G-protein-coupled receptor (GPCR) signaling. In contrast, the Turquoise module, which tracked with both GI symptoms and CARS scores, was dominated by a lipid-centric signature, encompassing PPARA-mediated regulation, omega-3/6 fatty acid synthesis, and primary bile acid biosynthesis. Meanwhile, the Green module appeared functionally distinct, focusing on protein translation and amino acid transport. These patterns imply that specific dysfunctions, particularly in lipid and bile acid regulation, may serve as molecular bridges connecting gut symptoms to the broader ASD pathology (Figures 4H,I; Supplementary Figure S2D).

### 3.6 Multi-omics integration and analysis

**Figure 5:**
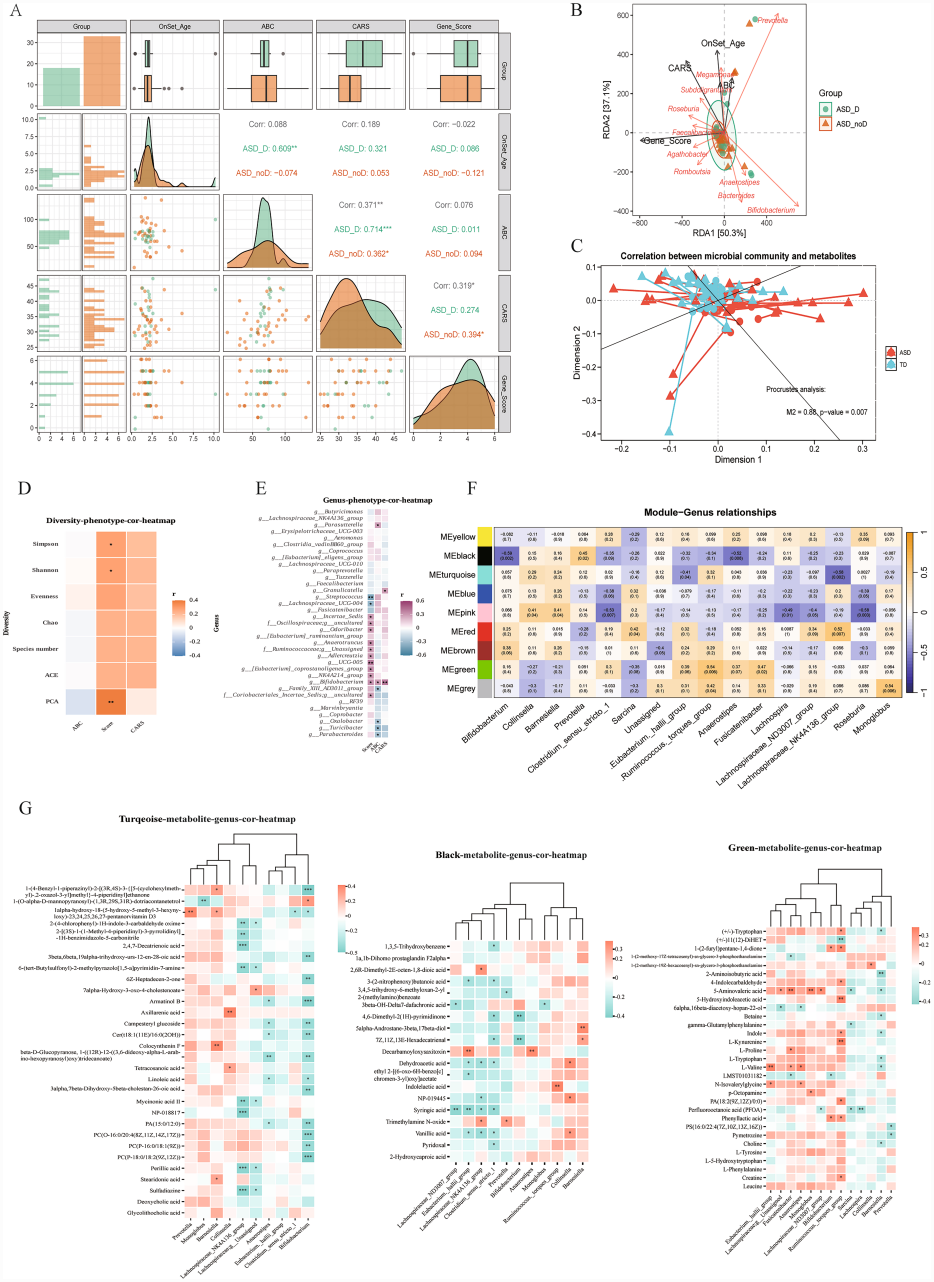
Multi-omics integration reveals complex interactions among host genetics, gut microbiota, and metabolism. **(A)** Multivariate scatter matrix displaying the distributions and pairwise correlations among clinical phenotypes (ABC, CARS, onset age) and the MUC gene variant score. The diagonal represents the data distribution, while the upper triangle shows the correlation coefficients. **(B)** RDA visualizing the influence of host factors (clinical traits and gene scores) on the gut microbial community structure. **(C)** Procrustes analysis demonstrating a significant global concordance between the gut microbiome and metabolome profiles. The short vertical lines connecting the points indicate a strong fit between the two datasets (M^2^ = 0.88, *p* = 0.007). **(D,E)** Spearman correlation heatmaps associating clinical severity (ABC and CARS scores) and MUC variant burden (gene score) with **(D)** microbial diversity indices and **(E)** specific bacterial genera. **(F)** Heatmap illustrating the relationships between WGCNA metabolic modules and key microbial genera, revealing functional microbe-metabolite associations. **(G)** Correlation heatmaps detailing the specific associations between metabolites within key functional modules (Turquoise, Black, and Green) and gut microbial taxa.

To investigate the interplay among host genetic background, clinical phenotypes, and the gut ecosystem, we performed an integrated analysis of MUC gene variant scores (Gene_Score), clinical data, and microbiome profiles in children with ASD. The results revealed a significant positive correlation between the MUC gene variant score and CARS score (r = 0.319, *p* < 0.05), suggesting that the accumulation of mucin-related gene mutations may be associated with disease severity. Furthermore, after stratifying by gastrointestinal symptoms (diarrhea), the correlations among clinical phenotypes (e.g., ABC vs. CARS) were notably more pronounced within the symptomatic group (ASD_D) compared to the asymptomatic group (Figure 5A). In the microbiome analysis, correlation analysis revealed a significant positive correlation between MUC variant scores and alpha diversity indices (Simpson and Shannon), as well as with the PCA results reflecting overall community structure (Figure 5D). This suggests that an elevated MUC gene variant burden may impair the integrity of the intestinal mucus barrier, which in turn may promote the proliferation of mucin-degrading bacteria and opportunistic pathogens, while reducing the abundance of beneficial bacteria such as butyrate-producing bacteria. This complex perturbation may manifest as an increase in microbial species richness, accompanied by functional imbalance and a decline in ecological stability. At the genus level, the MUC variant score exhibited specific positive correlations with mucin-utilizing or mucus-resident genera (e.g., *Bifidobacterium, Streptococcus, and certain [Eubacterium] groups*), while showing a negative trend with butyrate-producing, barrier-maintaining bacteria (e.g., Faecalibacterium and specific Lachnospiraceae subgroups) (Figure 5E). RDA analysis further confirmed that MUC gene scores, alongside clinical phenotypes (CARS, ABC), drivers of the variation in gut microbiota composition (Figure 5B). Collectively, these findings suggest that a high burden of MUC gene mutations may reshape the gut microecology in ASD by selectively enriching mucin-interacting bacteria while depleting barrier-protective taxa.

We further integrated gut microbiota profiles with plasma metabolites to examine the microbiome’s regulatory influence on metabolic patterns in children with ASD. Procrustes analysis revealed a significant overall concordance between microbial community structure and metabolic profiles (*p* = 0.007) (Figure 5C). Module-Genus correlation analysis based on WGCNA showed that the MEturquoise module was significantly negatively correlated with key butyrate-producing taxa, including Lachnospiraceae_NK4A136_group and Eubacterium_hallii_group. The green module demonstrated significant positive correlations with mucin-degrading or glycolytic taxa such as *Ruminococcus torques*_group and Fusicatenibacter. In addition, the black module exhibited the broadest range of microbial associations (Figures 5F,G).

## 4 Discussion

In this study, we integrated phenomics, genomics, microbiome, and metabolomics data from children with ASD to systematically explore the complex interactions among ASD severity, host genetic background, and gut microbial dysbiosis. Across multi-omics layers, our analyses collectively point to a potential mechanistic hypothesis: rare deleterious variants in the MUC gene pathway may be associated with intestinal mucus barrier impairment, which in turn correlates with the dysregulation of gut microbial ecosystem in ASD subjects. This barrier disruption leads to the depletion of butyrate-producing bacteria and broader microbial imbalance; in turn, the altered microbial community contributes to metabolic dysfunction, particularly the depletion of key metabolites such as butyrate. Collectively, these changes exacerbate gastrointestinal symptoms (e.g., diarrhea) and neurodevelopmental impairment (higher CARS scores). The identification of this “gene–microbiome–metabolism” axis offers new insights into the biological basis of ASD heterogeneity, especially gastrointestinal phenotypes, and provides potential avenues for personalized intervention.

### 4.1 Children with ASD exhibit pronounced gastrointestinal and metabolic dysregulation

We first confirmed the substantial clinical heterogeneity in our cohort, observing a high prevalence of GI symptoms that tracked with ASD severity. Consistent with widespread epidemiological evidence, we found that diarrhea was significantly associated with more severe autistic traits, reinforcing the pivotal role of gut dysfunction in ASD pathophysiology (Settanni et al., 2021; Fulceri et al., 2016). In parallel, our 16S rRNA profiling recapitulated the microbial signatures frequently reported in independent cohorts, including altered community structure and increased inter-individual variability (Ding et al., 2020; Zou et al., 2020; Xu et al., 2019).

Metabolomic profiling further demonstrated system-wide biochemical disturbances in ASD. Several pathways previously implicated in ASD were consistently altered, including perturbed glutamate–glutamine cycling (elevated Glu: Gln ratio), disrupted excitatory-inhibitory balance (increased GABA), urea-cycle and nitric oxide pathway abnormalities (altered arginine-, guanidinoacetate-, and proline-related metabolites), and reductions in phospholipid-related metabolites essential for neuronal membrane synthesis and cholinergic signaling (Wang et al., 2025; Dan et al., 2020). Together, these observations illustrate that children with ASD display coordinated alterations in gut microbial ecology and metabolic regulation.

In recent years, relevant studies have revealed that autism spectrum disorder (ASD) is consistently closely associated with gut microecological homeostasis imbalance and its associated metabolic abnormalities, despite variations in the specific microbial genera and metabolites identified across different research cohorts (De Sales-Millán et al., 2024; Xiang et al., 2025). Our findings are generally consistent with the core characteristics reported in previous literature, and further suggest that ASD-associated microbial and metabolic alterations may reflect a coordinated, systemic perturbation at the intestinal microenvironment level, rather than independent changes. Integrative multi-omics analysis provides a more unified interpretive framework for understanding the heterogeneous results observed in prior studies. However, the upstream host-driven mechanisms underlying this “gut-brain” association have remained unclear.

### 4.2 MUC variants as a novel upstream driver of dysbiosis

To address this gap, we performed an integrated analysis of genomic, microbiome, and metabolomics data. A key finding was that the genetic susceptibility of ASD extends beyond classical neurodevelopmental genes. Rare-variant enrichment analysis revealed significant aggregation of pathogenic variants in pathways related to O-glycosylation and immune regulation (e.g., Dectin-2 signaling and interferon-*γ*). These pathways converged on the MUC gene family, including *MUC21, MUC12, MUC17*, and *MUC6* genes, encoding mucins that are essential structural components of the intestinal mucus layer. Mucins are highly glycosylated proteins abundantly expressed on the gastrointestinal epithelium and within luminal secretions. Beyond providing a physical barrier, they actively shape host–microbiome interactions by regulating microbial adhesion, creating nutrient niches, and modulating epithelial immune responses (Dharmani et al., 2009; Lievin-Le and Servin, 2006). The O-glycosylation of mucins is particularly critical, as it determines mucin folding, viscoelastic properties, microbial binding specificity, and the stability of the mucosal barrier. Disruption of mucin glycosylation has been experimentally shown to impair epithelial integrity, increase susceptibility to inflammation, and alter gut microbial ecology (Wandall et al., 2021). Based on these findings, we hypothesized that the accumulation of rare harmful variants in MUC pathways represents an upstream host factor contributing to ASD-associated gut pathology by compromising the integrity of the epithelial mucus barrier.

Our multi-omics data are consistent with this hypothetical association. Clinically, higher MUC variant burden was associated not only with more severe ASD symptoms (higher CARS scores) but also with more pronounced GI manifestations (diarrhea/constipation), providing the first genetic-level evidence linking impaired mucus barrier function with ASD severity. Microbiologically, the MUC variation score was associated with significant shifts in gut microbial composition: higher MUC variant burden correlated with reduced levels of key butyrate-producing bacteria (e.g., Faecalibacterium), which are important for maintaining barrier integrity, and increased levels of mucin-utilizing taxa such as Bifidobacterium (Bottacini et al., 2014; Yamada et al., 2019). These observations suggest that host genetic defects in mucin biosynthesis drive microbial community restructuring by imposing strong selective pressure on the gut environment. These insights help explain why GI symptoms often track with ASD severity and implicate host genetics as a major upstream determinant.

### 4.3 The metabolic imprint of gut microbiome restructuring

Having identified host-driven microbial imbalance, we further investigated how these microbial changes impact metabolic function and clinical phenotypes. Procrustes analysis demonstrated a significant global correspondence between gut microbial composition and plasma metabolomic profiles, indicating that structural dysbiosis has functional metabolic consequences. To dissect this relationship, we performed WGCNA and identified several metabolite modules associated with both clinical features and specific microbial taxa.

The Turquoise module represented the most critical pathological axis. This module, enriched for bile acid and lipid transport metabolites, was positively correlated with abundant butyrate-producing taxa (e.g., Lachnospiraceae_NK4A136_group and Eubacterium_hallii_group) (Bunesova et al., 2018; Udayappan et al., 2016). Importantly, tissue enrichment analysis revealed that metabolites in this module were significantly associated with Cerebral Spinal Fluid (CSF) and Placenta. This finding is consistent with the module’s strong correlation with more severe neurodevelopmental impairment (higher CARS scores), further supporting a potential metabolic link to CNS function (Supplementary Figure S2A). Within the context of MUC pathway variation, we infer that the depletion of butyrate producers (such as Faecalibacterium) may lead to reduced levels of metabolites essential for gut homeostasis and lipid transport (e.g., linoleic acid, palmitic acid), thereby simultaneously aggravating gut symptoms and central nervous system dysfunction. The Green module was positively associated with mucin-degrading bacteria (e.g., *Ruminococcus torques* group) and glycolytic taxa (e.g., Fusicatenibacter). Metabolites in this module were enriched in Saliva and Epidermis (Supplementary Figure S2C). This tissue distribution aligns with the functional profile of the associated taxa (specifically mucin degradation), suggesting that a compromised mucus barrier may create ecological niches for the overgrowth of mucin-degrading microbes. Their metabolic activity may, in turn, perturb amino acid metabolism and contribute to ASD-related symptoms (Schaus et al., 2024). Although the Black module was not directly linked to clinical outcomes, its metabolites were enriched in feces and strongly associated with multiple bacterial taxa, further supporting the widespread influence of gut microbes on metabolic processes (Supplementary Figure S2B).

## 5 Limitations and conclusion

Despite these advances, the study has limitations. The use of 16S rRNA sequencing restricts functional resolution of the microbiome, and association-based analysis cannot establish causality. Future studies should combine metagenomic sequencing with experimental validation in animal models, such as MUC gene knockout mice and microbiota transplantation, to further elucidate causal pathways and evaluate therapeutic potential.

We included “sex” as a covariate in all multivariate analyses and confirmed core associations remained consistent across sex subgroups (*p* > 0.05), indicating no significant impact on study results.

In summary, our study validates and extends the current understanding of ASD pathophysiology by proposing a host-driven “gene–microbiome–metabolism” framework. Rare deleterious variants in MUC pathways appear to underlie impaired mucus barrier function and subsequently reshape microbial community structure. This genetically induced microbial imbalance leads to disturbances in key metabolic pathways, jointly contributing to both gastrointestinal symptoms and neurodevelopmental impairments. Thus, the MUC gene pathway may represent a critical upstream node for understanding ASD heterogeneity and a promising target for future precision stratification and individualized therapeutic strategies.

## Data Availability

The data involved in this study have been deposited in the Genome Sequence Archive (Genomics, Proteomics & Bioinformatics 2025) in National Genomics Data Center (Nucleic Acids Res 2025), China National Center for Bioinformation/Beijing Institute of Genomics, Chinese Academy of Sciences (GSA: CRA035219) that are publicly accessible at https://ngdc.cncb.ac.cn/gsa.
