# Supplementary material for: Integrated multi-omics analysis reveals the involvement of the gut-brain axis in children with autism
Source: Front Microbiol. 2026 Feb 4;17:1766850. doi: 10.3389/fmicb.2026.1766850 (PMC12913364; doi:10.3389/fmicb.2026.1766850)
Supplement: Supplementary file 1 [file Supplementary_file_1.docx]

**Integrated Multi-Omics Analysis Reveals the Involvement of the Gut-Brain Axis in Children with Autism**

**Contents**

**Supplementary methods**

**Supplementary Table S1. Clinical Manifestations of 51 age-matched TD controls.**

**Supplementary Table S2. ASD vs. TD: Demographics, Sleep and GI Disorders.**

**Supplementary Table S3. Detailed association statistics and functional annotations of rare deleterious variants identified in the ASD cohort.**

**Supplementary Figure S1. Comparative analysis of gut microbiota composition and diversity across groups and subgroups.**

**Figure S2 Tissue/Body Fluid Localization Enrichment of Metabolites Module (Top 25)**

**Supplementary methods**

1. **Genomic Profiling**

For WES, genomic DNA was extracted from peripheral venous blood of patients. High-qualify DNA libraries underwent sequencing on the Illumina NovaSeq 6000 platform, achieving an average depth of 100-200× and read lengths of 150 bp, covering over 95% of the target regions. Initial alignment was performed against the hg19/GRCh37 human reference genome using BWA software, followed by GATK-based detection of sequence variations including single nucleotide variants and indels. ANNOVAR facilitated subsequent variant annotation and filtration according to variant functionality, variant region, and variant impact.

1. **Microbil Profiling**

Fecal samples from children with ASD and matched controls were collected in sterile containers and immediately stored at –80 °C. Microbial genomic DNA was extracted using a standardized commercial kit according to the manufacturer's instructions. The hypervariable regions of the 16S rRNA gene were amplified by PCR with barcode-specific primers. PCR products were purified using Agencourt AMPure XP magnetic beads, and library quality was assessed with an Agilent 2100 Bioanalyzer. Qualified libraries were sequenced on the Illumina platform using paired-end reads.

Raw sequencing data were processed using a sliding-window approach to trim low-quality bases (window size 25 bp, average quality < 20). Sequences that were too short, contained adapters or ambiguous bases, or exhibited low complexity were discarded. High-quality reads were processed in QIIME2 for primer trimming, paired-end merging, and denoising to generate feature tables.

1. **Metabolomic Profiling**

**(1)** **Sample Preparation and Metabolite Extraction**
 Peripheral blood samples were collected from patients with ASD and TD, centrifuged, and stored at −80 °C. After thawing, samples were centrifuged at 4 °C and 3000 rpm to remove precipitates. A 100 μL aliquot of each sample was transferred into an EP tube and mixed with four volumes of methanol by vortexing, followed by incubation for 60 minutes at room temperature. The mixture was then centrifuged at 4 °C for 20 minutes, and the supernatant was collected and dried under vacuum using a freeze dryer. The dried residue was reconstituted in 100 μL of reconstitution solution, centrifuged again, and the supernatant was subjected to LC-MS analysis.

**(2) Mass Spectrometry Detection**
 An Accucore HILIC column was used for chromatographic separation. Column temperature was maintained at 40 °C with a flow rate of 0.3 mL/min. In positive ion mode, mobile phase A consisted of 0.1% formic acid, 95% acetonitrile, and 10 mM ammonium acetate, while mobile phase B consisted of 0.1% formic acid, 50% acetonitrile, and 10 mM ammonium acetate. In negative ion mode, formic acid was excluded from the mobile phases, and pH was adjusted to 9.0. The chromatographic gradient elution was performed according to a predefined program. Mass spectrometry was conducted in both positive and negative ionization modes, with a scan range of m/z 100–1500. ESI source settings were as follows: spray voltage, 3.2 kV; sheath gas flow rate, 3.5 arb; auxiliary gas flow rate, 10 arb; capillary temperature, 320 °C. MS/MS acquisition was performed in data-dependent scan mode.

**(3) Data Analysis**
 Raw data files were imported into Compound Discoverer 3.1 software for preprocessing. Retention time and mass-to-charge ratio (m/z) were filtered based on set thresholds. Peak detection, alignment, and integration were performed, and target ions were merged. Molecular formulas were predicted based on molecular and fragment ions, and compound identification was achieved by matching with reference databases. All data underwent quality control to ensure accuracy and reproducibility of the results.

**Supplementary Table S1. Clinical Manifestations of 51 age-matched TD controls.**

|  |
| --- |

| **Category** | **Subcategory** | **n (%) / Mean(Range)** |
| --- | --- | --- |
| **Demographics** | Male | 36 (70.6%) |
|  | Female | 15 (29.4%) |
|  | Age at enrolment (years) | 7 (5–8) |
|  |  |  |
| **Birth History** | **Gestational age** |  |
|  | < 37 weeks (Preterm) | 2 (3.9%) |
|  | > 42 weeks (Postterm) | 1 (2.0%) |
|  | 37–42 weeks (Term) | 48 (94.1%) |
|  | Mode of delivery |  |
|  | Vaginal delivery | 24 (47.1%) |
|  | Cesarean section | 27 (52.9%) |
|  | **Adverse birth history** |  |
|  | Macrosomia (> 4000 g) | 3 (5.9%) |
|  | Low birth weight (< 2500 g) | 4 (7.8%) |
|  | Nuchal cord | 3 (5.9%) |
|  | Amniotic fluid and meconium aspiration | 1 (2.0%) |
|  | Placental abnormality | 1 (2.0%) |
|  | Abnormal amniotic fluid | 3 (5.9%) |
|  |  |  |
| **Family Information** | **Parental age at conception (years)** |  |
|  | Father | 30.73 (28–32.5) |
|  | Mother | 30.40 (28.5–32) |
|  | Residential area |  |
|  | Urban | 48 (94.1%) |
|  | Town | 2 (3.9%) |
|  | Rural | 1 (2.0%) |
|  | **Annual household income (RMB)** |  |
|  | ＜ 50,000 | 5 (9.8%) |
|  | 50,000-100,000 | 12 (23.5%) |
|  | 100,000-500,000 | 33 (64.7%) |
|  | **Father’s educational level** |  |
|  | Junior high school or below | 10 (19.6%) |
|  | Senior high school to junior college | 21 (41.2%) |
|  | Bachelor’s degree | 20 (39.2%) |
|  | **Mother’s educational level** |  |
|  | Junior high school or below | 5 (9.8%) |
|  | Senior high school to junior college | 19 (37.3%) |
|  | Bachelor’s degree | 27 (52.9%) |
|  |  |  |
| **Behavioral Phenotype** | **Sleep disorders** |  |
|  | Insomnia | 1 (2%) |
|  | Hypersomnia | 1 (2%) |
|  |  |  |
| **Other Clinical Phenotypes** | **Gastrointestinal disorders** |  |
|  | Constipation | 2 (3.9%) |
|  | Diarrhea | 2 (3.9%) |

|  |
| --- |

|  |
| --- |
|  |

**Supplementary Table S2. ASD vs. TD: Demographics, Sleep and GI Disorders**

| **Category** | **Subcategory** | **n (%) / Mean(Range)** | | ***p*** |
| --- | --- | --- | --- | --- |
|  |  | **ASD** | **TD** |  |
| **Demographics** | Male | 36 (70.6%) | 36 (70.6%) | ＞0.99 |
|  | Female | 15 (29.4%) | 15 (29.4%) |  |
|  | Age at enrolment (years) | 7 (5-9) | 7 (5-8) | 0.85 |
|  | Age at onset (years) | 2.23 (1.59-2.34) | - |  |
|  |  |  |  |  |
| **Behavioral Phenotype** | **Sleep disorders** |  |  |  |
|  | Insomnia | 18 (35.3%) | 1 (2%) | ＜0.0001 |
|  | Hypersomnia | 11 (21.6%) | 1 (2%) | 0.0038 |
|  |  |  |  |  |
| **Other Clinical Phenotypes** | **Gastrointestinal disorders** | |  |  |
|  | Constipation | 36 (70.6%) | 2 (3.9%) | ＜0.0001 |
|  | Diarrhea | 18 (35.3%) | 2 (3.9%) | ＜0.0001 |

|  |
| --- |

**Supplementary Figure S1. Comparative analysis of gut microbiota composition and diversity across groups and subgroups.**

**
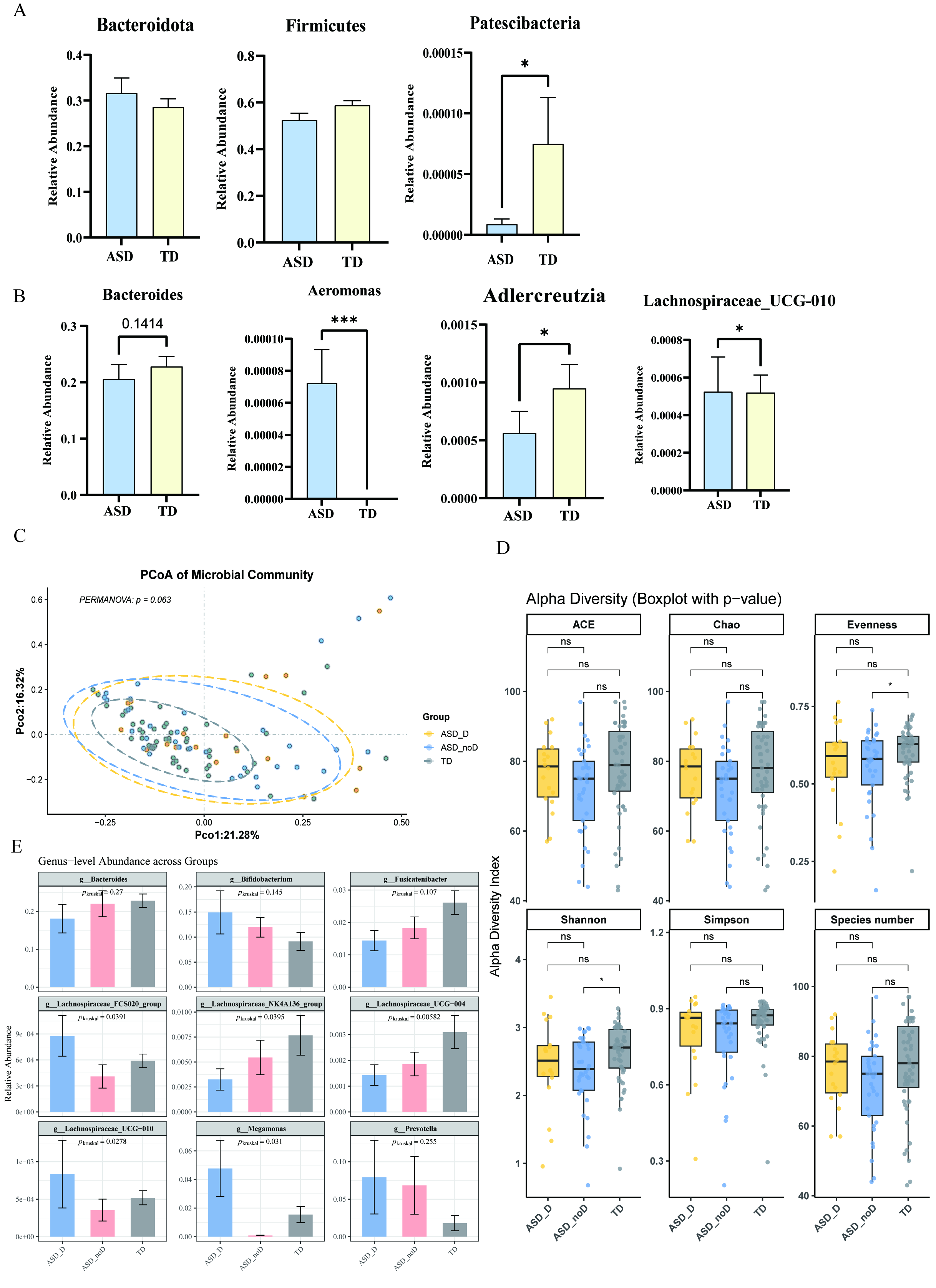
**

(A–B) Comparison of relative abundances of gut microbiota at the phylum (A) and genus (B) levels between the ASD and TD groups.

1. PCoA based on microbial community structure illustrating the distribution of beta diversity among the ASD with diarrhea (ASD_D), ASD without diarrhea (ASD_noD), and TD groups.
2. Comparison of alpha diversity indices (ACE, Chao, Evenness, Shannon, Simpson, and Species number) among the three groups.

(E) Differences in the relative abundance of specific bacterial genera across the three groups. (Data in bar charts are presented as mean ± SEM)

**Figure S2 Tissue/Body Fluid Localization Enrichment of Metabolites Module (Top 25)**


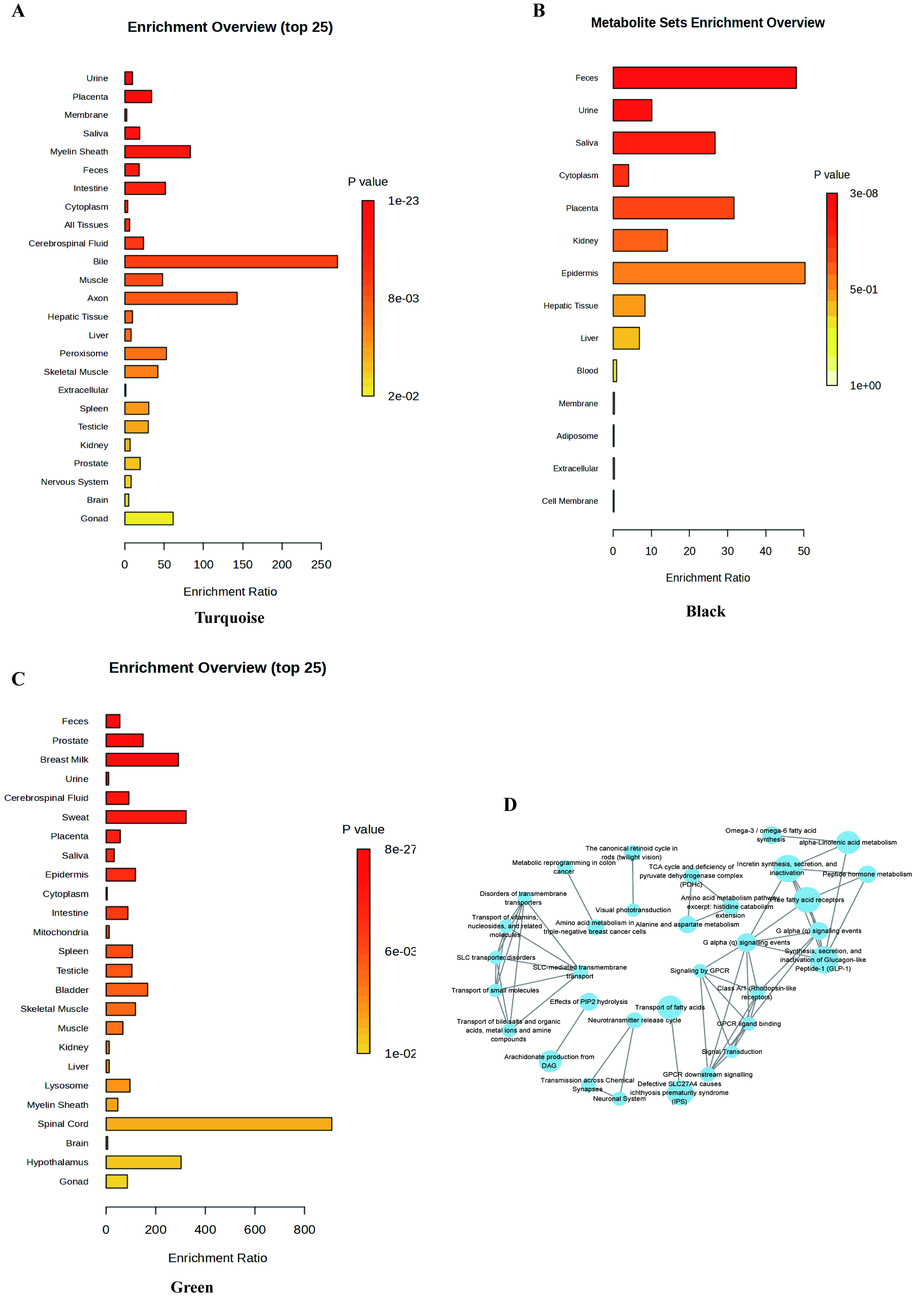


(A-C) Bar charts showing the top 25 tissue/body fluid localization enrichment results for metabolites in three modules: Turquoise module (A), Black module (B), and Green module (C). "Enrichment Ratio" indicates metabolite enrichment level at corresponding locations, calculated as Hits/Expected (hits = observed hits; expected = expected hits).

(D) Network visualization of the MEblue modules.
